# Controlling glutathione entry into mitochondria: potential roles for SLC25A39 in health and (treatment of) disease

**DOI:** 10.1038/s41392-022-00928-w

**Published:** 2022-03-09

**Authors:** Oliver von Bohlen und Halbach

**Affiliations:** Institute for Anatomy and Cell Biology, Friedrich Loeffler Str. 23c, 17489 Greifswald, Germany

**Keywords:** Cell biology, Molecular medicine

Members of the mitochondrial solute carrier (SLC) family 25 (SLC25) provide transport steps for substances across the mitochondrial inner membrane into the mitochondria that are needed for biochemical pathways and cellular homoeostasis. In a recent paper in Nature, Wang et al.^1^ could demonstrate that glutathione (GSH) transport seems to be mediated by SLC25A39, since loss of SLC25A39 reduces mitochondrial GSH import and abundance without affecting cellular GSH levels. Furthermore, they could show that cells lacking both SLC25A39 as well as its paralogue SLC25A40 exhibit defects in the activity and stability of proteins containing iron–sulfur clusters (ISCs).^1^ In addition, the results presented strongly support the view that mitochondrial GSH play essential roles for mediating ISC biogenesis as opposed to redox buffering.

**Fig. 1:**
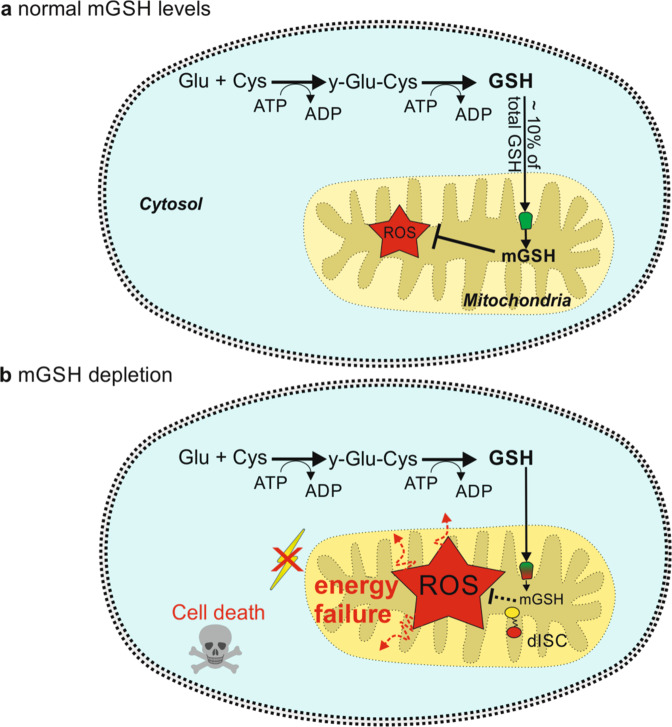
Glutathione (GSH) is synthesized in the cytoplasm by the action of γ-glutamylcysteine synthetase as well as glutathione synthetase, which both require ATP. About 10% of the synthesized GSH is transported into mitochondria under the participation of SLC25A39 (in green). **a** The GSH inside the mitochondria (mGSH) is involved e.g., in the detoxification of reactive oxygen species (ROS). **b** Impaired function of SLC25A39 has no major effect on the synthesis of GSH in the cytosol, but limits its transport into the mitochondria. Depletion of mGSH below a certain threshold can compromise ROS detoxification, leading to an accumulation of ROS within the cell. This in turn could induce energy failure as well as activation of caspases in the cytosol, resulting in cell death. Moreover, mGSH depletion impairs the stabilization and activation of proteins containing iron-sulfur clusters (disturbed ISCs = dISC)

Mitochondria are specialized membrane-bound organelles that play key roles in iron metabolism in that they synthesize heme, assemble iron-sulfur proteins, and are involved in the cellular iron regulation. Moreover, they generate most of the chemical energy (e.g., through the citric acid cycle and the electron transport chain) needed to power the cell’s biochemical reactions and, thereby, they are a source for free radicals. However, cell-damage could be induced by abundant production of free radicals that in turn can trigger malfunctions such as cancer or neurodegeneration. Cells could avoid or reduce this issue by synthesizing sufficient levels of antioxidants that are capable of neutralizing these free radicals. GSH is the major antioxidant that is produced within cells. Since GSH is produced in the cytosol but is also required by mitochondria, a transport into the mitochondria is needed. GSH in mitochondria is involved e.g., in the detoxification of reactive oxygen species (ROS; Fig. 1a).

Wang et al. have identified SLC25A39 as an important mitochondrial membrane carrier regulating GSH transport into mitochondria. Interestingly, a few years ago it has been published that in flies missense mutations of the SLC25A39 homologue “shawn” results in accumulation of ROS as well as in mitochondrial dysfunction, synaptic defects, and neurodegeneration.^2^ Since SLC25A39 protein is expressed in various tissues including the postnatal brain (see for details e.g.,: https://www.proteinatlas.org/ENSG00000013306-SLC25A39), this may hint for a specific role of that carrier in mitochondrial activity of certain neuronal populations. Malfunction of SLC25A39 might interfere with synaptic dysfunction or even neurodegeneration not only in flies but also in mammals, including humans. It has been speculated that oxidative stress as well as mitochondrial damage may act together in inducing neurodegeneration. Moreover, there is evidence that both, oxidative stress and mitochondrial damage are associated with changes in GSH homeostasis. Along this line, it has been hypothesized that decreased levels of GSH, which have been detected in post-mortem brains of parkinsonian patients at the cellular level, are likely to affect mitochondrial functions via loss of mitochondrial GSH.^3^ Parkinson’s disease (PD) is the second most common neurodegenerative disorder affecting about 1–5% of the general population. Loss of the neurotransmitter dopamine, due to the degeneration of dopaminergic neurons in the midbrain underlies the pathophysiology of the motor dysfunction. Until now, the cause of PD is unknown, but several factors seem to play a role, including environmental factors, such as the pesticide rotenone, that can block mitochondrial functions. In the last years, several genes have been identified to be involved in the etiopathogenesis of PD as well as genes that are associated with PD. Among these genes, some of them are associated with alterations of free radical formation or mitochondrial dysfunctions. In parallel to the finding of Wang et al., Gialluisi et al. have described the identification of novel candidate genes for late onset PD. Among them, several genes seemed to be involved in mitochondrial metabolism and oxidative stress, as e.g., SLC25A39.^4^ In addition, SLC25A39 expression in adult dopaminergic neurons of the midbrain could be demonstrated by the same authors.^4^ Thus, defects in SLC25A39 induce severe disturbances in the mitochondrial GSH-import machinery contributing to neuronal degeneration. However, to get further insight in the role of SLC35A39 in the brain, the generation of specific conditional Slc25a39 knockout mice that carry a deletion of Slc25a39 exclusively in neurons or in the central nervous system may further boost our understanding on the roles and in the importance of SLC25A39 in neuronal tissues.

Wang et al. also show that mitochondrial GSH play essential roles in mediating ISC biogenesis. ISCs are involved in electron transport, substrate binding, enzymatic catalysis and play critical roles in maintaining genomic stability since several DNA polymerases and helicases require interaction with ISCs for proper functioning. Thus, disturbances in mitochondrial ICS biosynthesis could contribute to failures in the supply of energy (which might result in cell death) or to cancer, since genomic instability is considered as a hallmark of cancer and a driver of neoplastic transformation processes.^5^ Mutations in SLC25A39 can alter mitochondrial GSH levels (Fig. 1b) and thereby interfere with ICS biosynthesis which in turn can have an impact on the development of cancer.

Taken together, the discovery made by Wang et al. represents an important step in understanding the roles and functions of SLC25A39 as well as in the importance of GSH as a scavenging antioxidant. The identification of SLC25A39 may lead to a better understanding of disease pathways linked to oxidative stress in the nervous system, as e.g., PD. In addition, the possibility to manipulate SLC25A39 and/or to regulate the amount of GSH that enters mitochondria also offers the opportunity to study the importance of mitochondrial GSH in more detail, especially in mitochondria-rich tissues like liver or kidney. Like other SLC25 family members, SLC25A39 might represent a biomarker for cancer. SLC25A39 as well as mitochondrial GSH may also represent promising drug targets for cancer, since manipulating SLC25A39 functioning or mitochondrial GSH in tumor cells may induce selective oxidative stress, leading to cell death. For getting more insight, the identification of factors that are capable of regulating SLC25A39 levels or mitochondrial GSH import that influence the activity and stability of proteins containing ISCs would be necessary.
